# Cone-beam computed tomographic investigation of the association between impacted mandibular third molars and the development of distal caries in adjacent second molars in a Chinese population

**DOI:** 10.1016/j.heliyon.2024.e40655

**Published:** 2024-11-22

**Authors:** Wenyuan Zhou, Zaidao Xiong, Juan Fan, Tao Yang, Yongchun Gu

**Affiliations:** Department of Dentistry and Central Lab, Ninth People's Hospital of Suzhou, Soochow University, Suzhou, 215200, China

**Keywords:** Cone-beam computed tomography (CBCT), Impacted mandibular third molar, Dental caries

## Abstract

To investigate the association between impacted mandibular third molars (IMTMs) and the prevalence of distal caries in adjacent mandibular second molars (MSMs) in a Chinese population, a retrospective analysis was conducted using cone-beam computed tomography (CBCT) images obtained from routine dental practice for various diagnostic purposes. The impaction patterns of the mandibular third molars were recorded using Winter's classification and Pell and Gregory's classification. The occurrence and severity of distal caries in MSMs were also documented. Chi-square tests and logistical regression analyses were used to assess the association between distal caries in the MSM and variables. A total of 552 scans were included, and the frequency of IMTMs was 64.5 % (647/1003). According to Winter's classification, the most common type of impaction was mesioangular impaction (45.5 %), followed by horizontal (31.2 %) and vertical impaction (17.5 %). Based on Pell and Gregory's classification, level B impaction (54.9 %) and class II impaction (57.5 %) were the most frequent. Compared to the non-impaction group, the frequency of distal caries in the MSMs for the impaction group was significantly higher (19.5 % [126/647] vs. 3.5 % [12/346]; *p* < 0.01). The prevalence of carious lesion in MSMs associated with IMTMs increased with age, peaking in the 31–35 years age group, followed by a decrease in the 36–40 years age group, and then rising again in patients aged above 40 years. Logistical regression analyses confirmed that the pattern of IMTMs significantly associated with distal caries in MSMs, and mesioangular angulation of IMTM (OR = 11.22), position A (OR = 8.37), a type I ramus relationship (OR = 2.70), and the 18–25 age group (OR = 2.62) were identified as significant risk factors (all *p* < 0.01). In conclusion, the present study demonstrates that the pattern of IMTMs contributes to the development of distal caries in adjacent MSMs. Clinicians should carefully consider these factors when evaluating the need for prophylactic extraction of IMTMs to prevent distal caries in MSMs.

## 1 Introduction

Third molars are considered impacted when they have not fully erupted into a functional occlusion within the expected time [1]. The prevalence of third molar impaction ranges widely from 23.5 % to 73.2 % across different studies and populations [[2], [3], [4], [5]], and the occurrence of impacted mandibular third molars (IMTMs) is significantly higher than that of impacted maxillary third molars [6]. IMTMs can predispose a variety of pathologies, including pericoronitis, root resorption, distal periodontal pockets and distal caries of the mandibular second molar (MSM), cysts, and halitosis [[7], [8], [9]]. They can also cause orthodontic and prosthetic problems, and temporomandibular joint disorders [10]. Due to the high occurrence rate of IMTMs and their associated pathologies, extraction of IMTMs is one of the most frequent procedures in oral surgery among young adults. However, although it is generally considered a safe procedure, several complications can occur during tooth extraction or in the postoperative period, including alveolitis, infection, postoperative bleeding, injury of the inferior alveolar nerve, and dental trauma to the adjacent second molars [[11], [12], [13], [14]]. Therefore, when determining whether to remove the IMTM as a prophylactic measure, it is essential for clinicians to gather sufficient anatomical information. In addition to this, several other factors must also be evaluated, including patient age, risk of pathology, the patient's oral hygiene, and caries risk, among others. Nevertheless, the prophylactic removal of an asymptomatic IMTM remains controversial, while some practitioners advocate for early intervention to prevent potential complications that may arise from delayed removal, others argue that extracting asymptomatic teeth unnecessarily exposes patients to risks [6].

IMTMs can be classified by the angulation of mandibular third molar (Winter's classification [15]), the depth of the impaction relative to the adjacent MSM, and the available space between the distal surface of the MSM and the anterior border of the ramus (Pell and Gregory's classification [16]). Previous scholars [8] also reported that the pattern of mandibular third molar impaction is associated with the development of distal caries in MSMs, which often remain unnoticed for a long period of time. The concealed site was unfavorable for diagnosis and accessing for restoration, while delay in the treatment of caries lesion in MSMs may cause early tooth loss. Moreover, the ethnicity and geographical regions of the subjects are also important influencing factors on the prevalence of IMTM, as well as the associated carious lesions [17,18]. Additionally, the status of the third molar (such as impaction or agenesis) can be an important dental trait in anthropology, which can be used for population comparisons. Nevertheless, research specific to Chinese populations remains limited, and several areas of controversy persist [6].

Periapical and panoramic radiographs are conventional imaging methods frequently used to assess the patterns of tooth impaction and carious lesions [[2], [3], [4], [5]]. However, these methods have inherent limitations as they provide only two-dimensional images, which can result in overlapping structures and image distortion. The advantages of dental cone-beam computed tomography (CBCT) over traditional periapical or panoramic radiography for diagnosis and treatment planning have been well established by previous studies [19]. CBCT, being three-dimensional, is currently a more reliable imaging technique for revealing anatomical details, the accurate relationships between structures, as well as detecting hidden pathologic conditions and anatomic variations, due to its high resolution and non-destructive nature [19,20]. The aim of this retrospective CBCT study was to investigate the association between IMTMs and the prevalence of distal caries in adjacent MSMs in a Chinese population.

## 2 Materials and methods

### 2.1 Subjects

The study protocol was reviewed and approved by the Ethics Committee of Suzhou Ninth People's Hospital (Issuing Number: KY2022–089-01). The committee waived the need for participant consent for this retrospective study. Dental CBCT data (DICOM format), previously obtained in the Department of Dentistry, Suzhou Ninth People's Hospital, China, from June 2017 to June 2024, were screened and assessed by a trained examiner with three years of experience in dentistry (*W*. *Z*.). All the examinations were performed for diagnostic reasons before complex oral surgeries or orthodontic treatment and adhere to the ALARA (as low as reasonably achievable) principle. The inclusion criteria were as follows: (1) gender and age were documented, with the age being at least 18 years old; (2) each subject had bilateral MSMs and at least a mandibular third molar. The exclusion criteria were as follows: (1) age under 18 years old; (2) CBCT images of insufficient quality to allow for accurate detection of IMTM patterns and dental caries severity; (3) MSMs with root canal fillings, posts, and crowns.

### 2.2 CBCT scanning

**Fig. 1:**
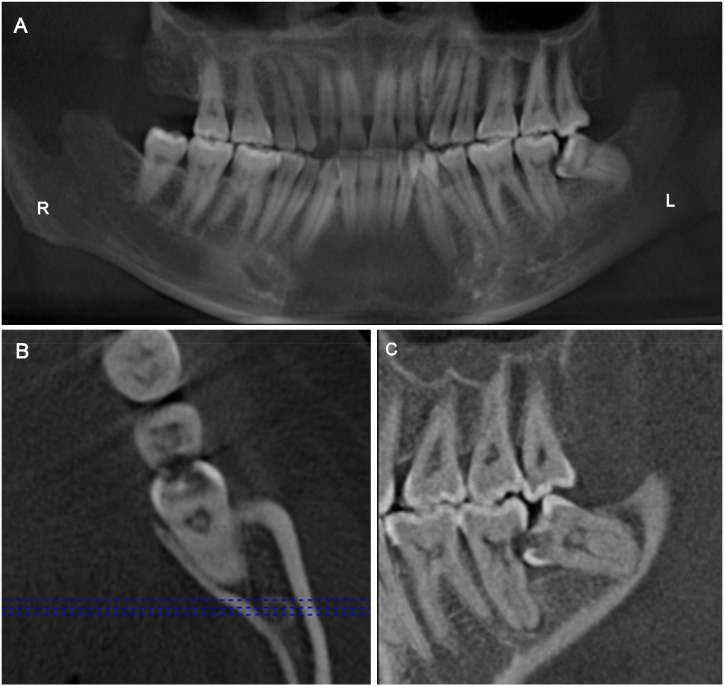
Representative CBCT images of an impacted mandibular third molar (IMTM). (A) The CBCT-generated panoramic image shows a mesioangular impaction on the left side, which is associated with carious lesions in both the mandibular second and third molars; (B) an axial section; (C) a sagittal section.

All dental CBCT examinations were performed using a 3D eXam i (KaVo Co., Germany) CBCT machine. The imaging protocol was as follows: field of view (FOV) = 14 × 8.5 cm; tube peak potential = 120 kVp; tube current = 5 mA; exposure time = 6 s; voxel size = 0.25 mm. The positions of the mandibular third molars as well as the severity of caries lesions in MSMs and mandibular third molars, were assessed by analyzing axial, sagittal, and coronal images, in addition to CBCT-generated panoramic images (Fig. 1A–C).

### 2.3 Classification of the position of the patched mandibular third molars

IMTMs can be classified using Winter's classification [15], based on their sagittal position, as vertical, mesioangular, horizontal, or distoangular. The angle between the long axis of the mandibular second and third molars is defined as follows: vertical (−10 to 10°), mesioangular (11–79°), horizontal (80–100°), and distoangular (−79 to −11°). According to Pell and Gregory's classification [16], which is based on the level of impaction, the impaction pattern is categorized into three classes: (a) Level A, where the occlusal plane of the mandibular third molar tooth is aligned with or positioned above the occlusal level of the adjacent MSM; (b) Level B, where the occlusal level of the mandibular third molar lies between cervical level and occlusal level of the adjacent MSM; (c) Level C, where the mandibular third molar is entirely buried in bone or located below the cement-enamel junction (CEJ) of the adjacent MSM. In considering the relationship of the IMTM to the ramus, the impaction pattern is divided into three classes: (a) Class I, where the third molar is located anterior to the anterior border of the mandible; (b) Class II, where the third molar is partially covered by the anterior border of the mandible; (c) Class III, where the crown of the third molar is completely covered by the anterior border of the mandible, and there is no space between the distal surface of the MSM and the anterior margin of the mandibular ramus.

### 2.4 Severity of caries lesion associated with IMTMs

**Fig. 2:**
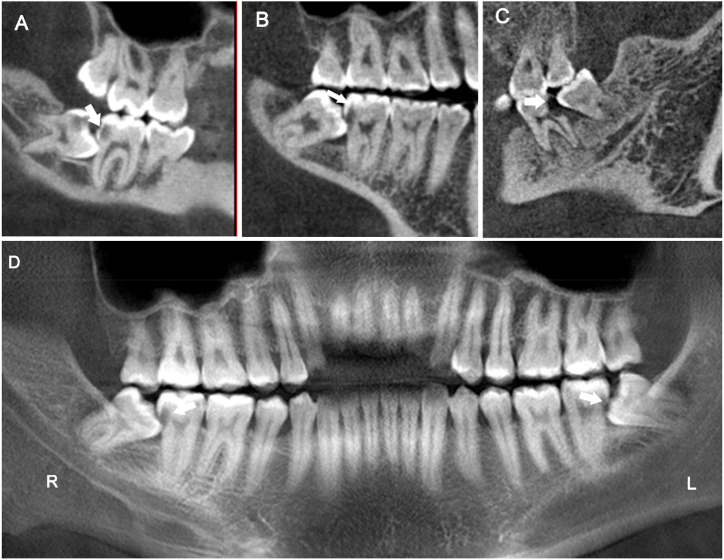
Classification of the severity of distal caries in mandibular second molars (CBCT images, arrows indicate distal caries in MSMs associated with IMTMs). (A) Shallow caries (sagittal section). (B) Moderate caries (sagittal section). (C) Deep caries (sagittal section). (D) The CBCT-generated panoramic image displays shallow caries in the left MSM and moderate caries in the right MSM.

The distal caries of MSM and the mesial caries of the mandibular third molar were classified into three categories according to the depth of the caries lesion (Fig. 2A–D): (A) shallow caries, where the caries lesion is confined to the enamel and/or the surface of the root; (B) moderate caries, where the lesion extends into the outer half of the dentine; and (C) deep caries, where the lesion penetrates the inner half of the dentine.

All assessments of IMTM patterns and associated carious lesions were performed by a single examiner (*W. Z.*). Calibration was conducted by an expert endodontist (*Y.G.*) and the examiner (*W.Z.*). In a pilot study, CBCT images from 50 subjects with bilateral mandibular second and third molars were used to evaluate the presence or absence of IMTM and distal caries in MSMs by both examiners. Disagreements were resolved through discussion, until a consensus was reached. Cohen's kappa test was used to evaluate the inter-observer and intra-observer errors. Each examiner independently evaluated the same 50 scans twice, with a 14-day interval between assessments.

Our pilot study found that the frequency of IMTM was 60 %. The incidence of distal caries in MSMs was 16 % in the IMTM group, compared to 5 % in the non-impaction group. Based on these data, a sample size calculation was performed using G∗Power (Version 3.1), assuming two-sided testing, α = 0.05, β = 0.20, and an allocation ratio of N2/N1 = 60/40 = 1.5. The analysis indicated that a minimum of 204 cases of MSMs/MTMs were required.

### 2.5 Statistical analysis

SPSS 24.0 software was used to analyze the data. The normality of the age data was assessed with the Shapiro-Wilk test, which indicated a statistically significant deviation from normality (*p* = 0.00). The Mann-Whitney *U* Test was employed to compare the medians between the two gender groups. A chi-square test was used to compare the frequencies. The association between distal caries in the MSMs and the variables was analyzed using both univariate and multivariate logistical regression analyses. The results were considered statistically significant if *p* < 0.05.

## 3 Results

In the detection of IMTMs, both the intra-observer and inter-observer kappa values were 0.86. For the detection of caries lesion in MSMs, the intra-observer kappa value was 0.89 (*W. Z*.), while the inter-observer kappa value was 0.94 (all *p* = 0.000). These results indicate that both inter-observer and intra-observer agreement were strong.

In total, 552 CBCT scans of subjects were selected based on the established inclusion and exclusion criteria, consisting of 272 males and 280 females. The median age of the subjects was 30 years, with an age range spanning from 18 to 70 years. No significant difference in age (*p* > 0.05) was found between the two genders.

The distribution of IMTMs is summarized in Table 1. Among a total of 1003 mandibular third molars, 647 teeth (64.5 %) were identified as IMTMs. The frequency was 66.1 % (329/498) on the right side and 63.0 % (318/505) on the left side, and the side difference was not statistically significant (*X*^2^ = 1.048, *p* = 0.306). The frequency of IMTMs was 68.3 % (338/495) in males, which was significantly higher (*X*^2^ = 8.587, *p* = 0.003) than the 60.8 % (309/508) observed in females. The most common type of impaction (by Winter's classification) was mesioangular impaction, accounting for 45.6 % (295/647) of all IMTMs, followed by horizontal (31.2 %) and vertical impactions (17.5 %). As classified by the Pell and Gregory's classification, level B impaction and class II impaction were the most common, with proportions of 54.9 % and 57.5 %, respectively.

**Table 1:** The distribution of impacted mandibular third molars (IMTMs) *n* (%).

Classification of Impaction	Male			Female			Male + Female		
	Right	Left	R + L	Right	Left	R + L	Right	Left	R + L
Winter's classification									
Vertical	26(10.6)	18(7.2)	44(8.9)	40(15.9)	29(11.3)	69(13.6)	66(13.3)	47(9.3)	113(11.3)
Mesioangular	78(31.7)	65(26.1)	143(28.9)	67(26.6)	85(33.2)	152(29.9)	145(29.1)	150(29.7)	295(29.4)
Horizontal	64(26.0)	67(26.9)	131(26.5)	36(14.3)	35(13.7)	71(14.0)	100(20.1)	102(20.2)	202(20.1)
Distoangular	5(2.0)	10(4.0)	15(3.0)	6(2.4)	5(2.0)	11(2.2)	11(2.2)	15(3.0)	26(2.6)
Others	4(1.6)	1(0.4)	5(1.0)	3(1.2)	3(1.2)	6(1.2)	7(1.4)	4(0.8)	11(1.1)
Impaction	177(72.0)	161(64.7)	338(68.3)	152(60.3)	157(61.3)	309(60.8)	329(66.1)	318(63.0)	647(64.5)
Non-impaction	69(28.0)	88(35.3)	157(31,7)	100(39.7)	99(38.7)	199(39.2)	169(33.9)	187(37.0)	356 (35.5)
Total	246 (100.0)	249 (100.0)	495 (100.0)	252 (100.0)	256 (100.0)	508 (100.0)	498 (100.0)	505 (100.0)	1003 (100.0)

**Pell and Gregory classification (Occlusal level)**									
A	67(37.9)	53(32.9)	120(35.5)	59(38.8)	60(38.2)	119(38.5)	126(38.3)	113(35.5)	239(36.9)
B	91(51.4)	95(59.0)	186(55.0)	85(55.9)	84(53.5)	169(54.7)	176(53.5)	179(56.3)	355(54.9)
C	19(10.7)	13(8.1)	32(9.5)	8(5.3)	13(8.3)	21(6.8)	27(8.2)	26(8.2)	53(8.2)
Total	177	161	338	152	157	309	329	318	647

**Pell and Gregory classification (Ramus relationship)**									
I	77(43.5)	52(32.3)	129(38.2)	81(53.3)	63(40.1)	144(46.6)	158(48.0)	115(36.2)	273(42.2)
II	99(55.9)	109(67.7)	208(61.5)	71(46.7)	93(59.2)	164(53.1)	170(51.7)	202(63.5)	372(57.5)
III	1(0.6)	0(0.0)	1(0.3)	0(0.0)	1(0.6)	1(0.3)	1(0.3)	1(0.3)	2(0.3)
Total	177	161	338	152	157	309	329	318	647

R is right; L is left. A total of 272 male and 280 female subjects were included.

The distribution of distal caries in MSMs and mesial caries in mandibular third molars associated with IMTMs is shown in Table 2. Regarding distal caries in MSMs, the frequency among all IMTM cases was 19.8 % (67/338) in males and 19.1 % (59/309) in females, with no statistically significant gender difference (*X*^2^ = 0.055, *p* = 0.815). Compared to the non-impaction group, the prevalence of distal caries in MSMs was significantly higher in the impaction group (19.5 % [126/647] vs. 3.4 % [12/356]; *X*^2^ = 50.189, *p* < 0.001). Conversely, for mesial caries in mandibular third molars, the frequency in the impaction group was only 9.4 % (61/647), closely resembling 11.5 % (41/356) in the non-impaction group (*X*^2^ = 1.097, *p* = 0.295), but significantly lower (*X*^2^ = 26.410, *p* < 0.001) than the 19.5 % observed in the adjacent MSMs.

**Table 2:** The prevalence of distal caries in M2 and mesial caries in IMTMs *n* (%).

Classification of impaction	Male			Female			Male + Female		
	*n* (R + L)	M2	M3	*n* (R + L)	M2	M3	*n* (R + L)	M2	M3
Winter's classification									
Verticle	44	3	6	69	2	4	113	5(4.4)	10(8.8)
Mesioangular	143	46	17	152	41	18	295	87(29.5)	35(11.9)
Horizontal	131	18	9	71	15	6	202	33(16.3)	15(7.4)
Distoangular	15	0	1	11	1	0	26	1(3.8)	1(3.8)
Others	5	0	0	6	0	0	11	0(0.0)	0(0.0)
Total IMTMs	338	67	33	309	59	28	647	126(19.5)	61(9.4)
Non-impaction	157	5	12	199	7	29	356	12(3.4)	41(11.5)
Total	495	72	45	508	66	57	1003	138(13.8)	102(10.2)

**Pell and Gregory's classification (Occlusal level)**									
A	120	39	19	119	33	20	239	72(30.1)	39(16.3)
B	186	25	14	169	26	8	355	51(14.4)	22(6.2)
C	32	3	0	21	0	0	53	3(5.7)	0(0.0)
Total IMTMs	338	67	33	309	59	28	647	126(19.5)	61(9.4)

**Pell and Gregory's classification (Ramus relationship)**									
I	129	43	22	144	35	20	273	78(28.6)	42(15.4)
II	208	24	11	164	24	8	372	48(12.9)	19(5.1)
III	1	0	0	1	0	0	2	0(0.0)	0(0.0)
Total IMTMs	338	67	33	309	59	28	647	126(19.5)	61(9.4)

M2 is distal caries in mandibular second molars; M3 is mesial caries in mandibular third molars.

R is right and L is left.

A total of 272 male and 280 female subjects were included.

Table 3 shows that the prevalences of caries lesions in mandibular second and third molars increase with age, reaching an initial peak in the 31–35 year age group. The frequencies then decrease in the 36–40 year age group, subsequently rising again among patients aged over 40 years.

**Table 3:** Influence of age on distal caries in mandibular second molars and mesial caries in impacted mandibular third molars *n* (%).

Age group	Mandibular second molars					Mandibular third molars			
(years)	*n*	Shallow	Moderate	Deep	Total	Shallow	Moderate	Deep	Total
Male									
18–25	75	4	1	2	7(9.3 %)	1	1	2	4(5.3 %)
26–30	97	4	8	2	14(14.4 %)	2	0	2	4(4.1 %)
31–35	112	15	9	9	33(29.5 %)	6	2	11	19(17.0 %)
36–40	32	2	4	1	7(21.9 %)	0	1	2	3(9.4 %)
>41	22	2	0	4	6(27.3 %)	0	1	2	3(13.6 %)
Total	338	27	22	18	67(19.8)	9	5	19	33(9.8 %)

**Female**									
18–25	115	14	2	1	17(14.8 %)	1	0	0	1(0.9 %)
26–30	82	8	2	4	14(17.1 %)	3	2	1	6(7.3 %)
31–35	63	12	2	1	15(23.8 %)	4	1	5	10(15.9 %)
36–40	28	0	2	2	4(14.2 %)	0	0	2	2(7.1 %)
>41	21	2	3	4	9(42.9 %)	0	2	7	9(42.9 %)
Total	309	36	11	12	59(19.1 %)	8	5	15	28(9.1 %)

**Male + Female**									
18–25	190	18	3	3	24(12.6 %)	2	1	2	5(2.6 %)
26–30	179	12	10	6	28(15.6 %)	5	2	3	10(5.6 %)
31–35	175	27	11	10	48(27.4 %)	10	3	16	29(16.6 %)
36–40	60	2	6	3	11(18.3 %)	0	1	4	5(8.3 %)
>41	43	4	3	8	15(34.9 %)	0	3	9	12(27.9 %)
Total	647	63	33	30	126(19.5 %)	17	10	34	61(9.4 %)

Univariate and multivariate logistical regression analysis demonstrated that the presence of distal caries in MSMs was significantly associated with IMTMs in position A (odds ratio [OR] = 8.37; *p* < 0.01), mesioangular angulations (OR = 11.22; *p* < 0.001), and type I ramus relationship (OR = 2.01; *p* < 0.01). Moreover, patients 18–25 years old were more likely to be affected by distal caries in MSMs (OR = 2.62; *p* < 0.01) (Table 4).

**Table 4:** Logistic regression models for distal caries in MSMs associated with variables.

Variables	Univariate		Multivariate	
	OR (95 % CI)	*P* value	OR (95 % CI)	*P* value
**Gender**				
female	1.05(0.71–1.55)	0.816	–	–
male	1			
**Side**				
left	1.12(0.76–1.66)	0.561	–	–
right	1			
**Age (yrs.)**				
18–25	2.32(1.25–4.30)	0.007	2.62(1.33–5.15)	0.005
25–30	1.82(1.00–3.32)	0.050	1.67(0.86–3.24)	0.129
31–35	0.89(0.51–1.54)	0.670	0.91(0.48–1.70)	0.760
>36	1			
**Winter's classification**				
mesioangular	9.97(4.24–23.42)	0.000	11.22(4.65–27.06)	0.000
horizontal	4.68(1.91–11.49)	0.001	7.01(2.75–17.86)	0.000
verticle + distoangular + other	1			
**Pell and Gregory's classification (Occlusal level)**				
A	7.04(2.13–23.33)	0.001	8.37(2.42–29.01)	0.001
B	2.75(0.83–9.15)	0.099	2.96(0.86–10.27)	0.087
C	1			
**Pell and Gregory's classification (Ramus relationship)**				
I	2.70(1.81–4.03)	0.000	2.01(1.27–3.17)	0.003
II + III	1			

## 4 Discussion

The assessment of the pattern of IMTMs in relation to pathological conditions is essential for clinicians to optimize patient management and determine the appropriate treatment plan. Additionally, variations in ethnicity and gender may influence the status of third molars, which could also have significant anthropological and forensic implications [21]. This retrospective CBCT study specifically investigates the prevalence of distal caries in MSMs associated with IMTMs within a Chinese population. This population is characterized by distinct dietary habits, oral health practices, and genetic factors, which may differ from those observed in other ethnic groups across various geographic regions [22].

Most previous studies have predominantly utilized panoramic radiographs [2,23] or periapical radiographs [24], both of which are two-dimensional imaging techniques. These methods inherently suffer from limitations such as anatomic noise, image overlap, and distortion, which can compromise diagnostic accuracy and assessment precision. In contrast, CBCT is widely regarded as the gold-standard imaging technique for evaluating the three-dimensional positioning of impacted teeth, as well as for accurately evaluating the location and severity of carious lesions-even in their early stages [19]. CBCT offers superior diagnostic precision by providing detailed, high-resolution images that allow for a comprehensive analysis of dental structures, free from the confounding factors associated with two-dimensional imaging. Furthermore, CBCT images have been shown to improve the detection and depth assessment of proximal and occlusal carious lesions [25].

Previous scholars have reported that the prevalence of IMTM generally ranged from 30.3 % to 68.6 % [[26], [27], [28]]. In this study, the overall prevalence of IMTM was found to be 64.5 %. This percentage is notably higher than the 42.6 %, reported in another Northeastern Chinese population [29]. However, it is lower than the 90 % observed in a Singapore Chinese population [30] and the 75 % reported in a Hong Kong Chinese population [31]. In this study, males exhibited a higher frequency of IMTM (68.3 %) compared to females (60.8 %), which contrasts with the findings of Alsaegh et al. [2], Rashid et al. [28], and Jaroń et al. [32], who reported a higher prevalence among females. Conversely, many other researchers [4,5,33,34] observed no significant gender predilection in the prevalence of IMTMs. The variations observed across studies may be attributed to differences in third molar mineralization rates, tooth-to-jaw relationships, and the rates of physical maturity among different populations [2]. Additionally, factors such as ethnic background, methodologies (including subject selection bias and sample size), and environmental influences may significantly impact these findings. Understanding these differences is crucial for interpreting the incidence of IMTMs in diverse populations and for tailoring clinical approaches accordingly.

In terms of the impaction pattern, as presented in Table 1, mesioangular impaction was the most common angular position, accounting for 45.6 % of all 647 IMTMs. This percentage is higher than that reported by Padhye et al. [33] (35 %) and Hugoson et al. [35] (30 %), but lower than that reported by Rashid et al. [28](51.1 %), Quek et al. [30] (59.5 %) and Kruger et al. [36] (62.9 %). It is, however, very close to the 47.4 % reported by Alsaegh et al. [2]. In regard to the impaction level, the results indicated that Level B (54.9 %) was the most prevalent, which aligns with the findings of Alsaegh et al. [2], Rashid et al. [28], and Padhye et al. [33]. However, several other studies reported that a higher impaction level (Level A) was the most common [32]. Table 3 shows that the prevalence of distal caries of MSMs decreased with the depth of IMTMs. Level C was the least common impaction pattern (8.2 %), and because the crown is completely embedded in bone, it is less prone to the development of a carious lesions. In contrast, the superior location of the third molar may result in a greater probability of food impaction and tooth decay. The mesiodistal space between the distal surface of the MSM to the ascending ramus of the mandible is an important variable to predict the eruption of third molars, as a lack of mesiodistal space in the jaw (< the mesiodistal width of the crown) may contribute to abortive eruption (Class II and III impactions) [37]. In the current study, Class II impaction (57.5 %) was the most common pattern. However, adequate mesiodistal space does not necessarily guarantee normal eruption [38], and Class I impaction accounted for a significant proportion of 48 %, indicating that the etiology of IMTMs is complex and associated with multiple factors.

Previous studies utilizing periapical and panoramic radiographs have demonstrated that the prevalence of caries in MSMs associated with IMTMs ranges from 13.4 % to 30.1 % [[39], [40], [41], [42], [43], [44]]. Kang et al. [45] conducted a CBCT study on a Chinese population and reported a significant higher prevalence of 52 % among all subjects with IMTMs; however, in the current CBCT study, the percentage was only 29.5 % in the impaction group, and 19.5 % among all subjects. The discrepancy may be due to the fact that, in Kang et al.’s study, all subjects underwent CBCT projection for preoperative assessment for the removal of MTMs. In Chinese populations, many patients do not seek dental services until their teeth exhibit symptoms, leading to a higher likelihood of including MSMs with distal caries [45]. In this study, neither side differences nor gender differences have been detected, which is consist with many other studies [2,7,28,46]; however, Syed et al. [23] reported a male predominance in a Saudi Arabian population (56.4 % in males vs. 43.6 % in females), and a similar finding was reported by Kang et al. [45] (60.6 % in males vs. 45.4 % in females) in a Chinese population. Conversely, Kunwar et al. [42] and several other scholars [43] reported that females had more caries in MSMs associated with IMTMs than males.

Regarding the impaction angulation, the highest prevalence of caries in MSMs was observed in the mesioangular impaction, which aligns with most previous reports [4,18,23,28,[46], [47], [48], [49]]. This suggests that the mesioangular impaction is an important risk factor for causing distal caries of MSMs. Kang et al. [45] reported that the angulation of the mandibular third molar was more stable and reliable indicator than the cement-enamel junction (CEJ) distance between the distal MSM and the mesial mandibular third molar in estimating risk factors related to the IMTM. They noted that mesioangular impaction within a range of 43°–73° might facilitate more food impaction and consequent carious lesions. For mesioangular or horizontal impactions, the occlusal surfaces of the third molars form plaque-accumulative crevices against the distal surfaces of the MSMs [45]. The pericoronal flap in this area impaired routine cleaning, and gingival recession may aggravate food impaction and exposure of cementum, thereby accelerating the initiation and progress of carious lesions [50]. Table 2 shows that Level A and Class I impactions are also associated with a higher prevalence of caries in the MSMs (30.1 % and 28.6 %, respectively) as compared with the other impaction categories, which is consistent with many other studies [46,47,49]; conversely, Level C and Class III impactions are often not in direct communication with the oral cavity, and thus, are less prone to bacterial plaque retention and caries development. The frequency of distal caries of MSMs in impaction group was significantly higher than in non-impaction group (29.5 % vs. 3.5 %), which is consistent with the findings of Skitioui et al. [4], indirectly confirming the view that distal caries in MSM can be caused by IMTM. Univariate and multivariate logistic regression analyses confirmed that IMTMs with mesioangular angulations (OR = 11.22), in position A (OR = 8.37), and in a type I ramus relationship (OR = 2.70) are significant risk factors that can markedly increase the likelihood of developing carious lesions in MSMs (Table 4). Collectively, the data presented in this report suggest that the impaction pattern may influence the occurrence of distal caries in adjacent MSMs. As a partially erupted tooth does not participate mastication, the concealed space and poor contacting point between the IMTM and MSM tend to favor the accumulation of bacterial plaque and debris. This accumulation cannot be effectively cleaned by either patients or professionals. Detection and restoration of distal caries in an MSM are often very challenging, and such teeth often end up being extracted [51].

IMTMs are often surgically removed due to pericoronitis, periodontitis, or caries lesions [52]. This study identified a higher prevalence of distal caries in MSMs compared to mesial caries in IMTMs (19.3 % vs. 9.4 %), likely attributed to the earlier eruption of MSMs. Furthermore, carious lesions in MSMs frequently occur near the distal cervical line, which is commonly associated with abnormal contact from mesioangular impactions. Compared to the mesial aspect of the mandibular third molar, the distal cervical region of the MSM appears more susceptible to caries initiation and development. This may be due to the thinner enamel and cementum layers near the distal cervical line of the MSM, rendering it more vulnerable to decay.

The prevalence and severity of distal caries in MSMs have been reported to increase with age in some studies [38,44]. However, Syed et al. [23] found that older age groups exhibited a lower prevalence of distal caries in MSMs. In contrast, Skitioui et al. [4] observed a peak prevalence in individuals aged 26–35 years, followed by a decline with further aging. According to Rashid et al. [28], logistic regression analysis revealed a significant association between distal caries in MSMs and the 19–27 age group (OR = 2.77) within a Kurdish population in Iraq; however, no such association was identified for the 28–65 age group. The discrepancies in these findings may be attributed to the progression of carious lesions over time, which ultimately affects the pulp, leading to root canal treatment or extraction of the affected teeth. Consequently, such teeth may be excluded from the study. In this study, the prevalence of caries in MSMs increased with age from 18 years, and reached the first peak in the age group of 31–35 years (29.5 % in males and 23.8 % in females); then decreased to 22.6 % in males and 14.6 % in females in the age group of 36–40 years, and finally, increased again in those over 40 years old. The increasing tendency was more prominent in females (with the prevalence reaching 42.9 % compared to 26.1 % in males). This finding further confirms the gender difference in the progression of carious lesions associated with IMTMs. The downward trend in the age group of 36–40 years may be due to the exclusion of many of cases with endodontically treated or extracted MSMs; after 40 years of age, this trend reversed, and an increase in carious lesions regained predominance. In this study, logistic regression analyses revealed a significant association between distal caries in MSMs and the 18–25 age group (OR = 2.62), whereas no significant association was identified for other age groups, consistent with the findings of Rashid et al. [25]. This warrants further investigation using an unbiased sampling method and a larger sample size. Overall, the current data suggest that prophylactic surgical removal of the IMTM should be considered for patients younger than 30 years old (before the first peak prevalence) to reduce the high risk of developing caries in adjacent MSMs.

One limitation of this study is that it is a retrospective study, and all the subjects were patients of a general hospital who underwent dental CBCT examination, which could lead to a biased sample in terms of disease spectrum. A prospective study design with long-term follow-up can overcome this limitation. Moreover, in comparison with other studies, the impaction classifications and methodologies used may be diverse (for example, some studies included all third molars, while other studies restricted their focus to mandibular third molars only). Under these circumstances, clinicians should be aware that direct comparisons of these studies could be misleading. Finally, the occurrence of IMTMs and carious lesions is influenced by multiple factors such as ethnicity, diet, the frequency and intensity of mastication, and genetic background. To verify a more reliable association between IMTM and caries of MSMs, further studies are required based on larger sample sizes of subjects and populations with diverse ethnic backgrounds.

## 5 Conclusions

This CBCT study highlights the substantial impact of IMTMs on the development of distal caries in adjacent MSMs within a Chinese population. Mesioangular angulation of IMTM (OR = 11.22), position A (OR = 8.37), a type I ramus relationship (OR = 2.01), and the 18–25 age group (OR = 2.62) are identified as significant risk factors. These findings suggest that clinicians should carefully consider these factors when evaluating the need for prophylactic extraction of IMTMs to prevent distal caries in MSMs.

## CRediT authorship contribution statement

**Wenyuan Zhou:** Writing – original draft, Methodology, Investigation, Data curation. **Zaidao Xiong:** Investigation, Formal analysis, Data curation. **Juan Fan:** Methodology, Investigation, Formal analysis. **Tao Yang:** Supervision, Conceptualization. **Yongchun Gu:** Validation, Supervision, Funding acquisition, Conceptualization.

## Ethic statement

This study was reviewed and approved by the Institutional Review Board of the Ninth People's Hospital of Suzhou, Suzhou, China, with the approval number: [KY2022-089-01].

## Additional information

No additional information is available for this paper.

## Data availability

Data included in article.

## Funding statement

This work was supported by Suzhou “Medical Health Science and Technology Innovation” Project [SKY2022030].

## Declaration of competing interest

The authors declare that they have no known competing financial interests or personal relationships that could have appeared to influence the work reported in this paper.
